# Physical exercise and depressive symptoms among older adults: a serial mediation model of self-rated health and subjective wellbeing

**DOI:** 10.3389/fpsyg.2026.1877978

**Published:** 2026-09-25

**Authors:** Run Yuan, Qian Wu, Quan Guo

**Affiliations:** 1 School of Physical Education, Shaoxing University, Shaoxing, China; 2 Business School, Shaoxing University, Shaoxing, China; 3 Shaoxing Local Legislation Research Center, Shaoxing, China

**Keywords:** active aging, depressive symptoms, older adults, physical exercise, serial mediation model

## Abstract

**Objective:**

Physical exercise is an important health-promoting behavior for older adults, while depressive symptoms are a major psychological factor affecting their quality of life and active aging. This study aimed to examine the associations between physical exercise and depressive symptoms among older adults and to further explore the potential indirect pathways involving self-rated health and subjective wellbeing.

**Methods:**

Using data from the 2023 Chinese General Social Survey (CGSS), a total of 2,233 older adults aged 60 years and above were included in the analysis. Multiple linear regression and serial mediation analyses were conducted using the PROCESS macro in SPSS. Bootstrap sampling with 5,000 resamples was applied to test the significance of the mediating effects.

**Results:**

Physical exercise was significantly and negatively associated with depressive symptoms among older adults, indicating that higher frequencies of exercise participation were associated with lower levels of depressive symptoms. In addition, physical exercise showed significant indirect associations with depressive symptoms through self-rated health (indirect effect = 40.5%) and subjective wellbeing (indirect effect = 9.7%), as well as through the serial pathway of “self-rated health → subjective wellbeing” (indirect effect = 3.8%). The total indirect effect (54%) was larger than the direct effect (46%), suggesting that a substantial proportion of the observed association between physical exercise and depressive symptoms was statistically accounted for by the indirect pathways examined in the model.

**Conclusion:**

Higher frequency of physical exercise was associated with lower levels of depressive symptoms among older adults, both directly and indirectly through self-rated health and subjective wellbeing. These findings highlight the potential relevance of physical exercise, self-rated health, and subjective wellbeing to mental health and active aging among older adults. Policies and interventions may consider strengthening support systems for older adults’ participation in physical exercise, while simultaneously addressing both physical and psychological wellbeing.

## 1 Introduction

With the continuous deepening of global population aging, mental health issues among older adults have attracted increasing attention worldwide. According to the United Nations, the global population is projected to reach 10.3 billion by 2080 (United Nations, 2024). China officially entered a deeply aging society in 2021 and has become one of the countries with the largest older adult populations in the world. According to the 2024 National Report on the Development of Aging, by the end of 2024, the population aged 60 years and above in China had reached 310 million, accounting for 22% of the total population. Against this demographic background, depression has become one of the most common negative psychological experiences among older adults (World Health Organization [WHO], 2025a; World Health Organization [WHO], 2025b). Depression not only reduces life satisfaction and quality of life among older adults (Viana Campos et al., 2014), but is also closely associated with cognitive decline (Zheng et al., 2018) and the aggravation of chronic diseases (Gao et al., 2026). Therefore, identifying factors associated with lower depressive symptoms among older adults is of practical significance for promoting mental health, quality of life, and active aging.

Physical exercise, as an important health-promoting behavior, has been consistently associated with better mental health among older adults. Previous studies have shown that regular physical exercise is associated with lower levels of depressive symptoms among older adults (Wang and Ren, 2022; Gao et al., 2025). Beyond the absence of disease, healthy and active aging also encompasses quality of life, emotional wellbeing, social participation, and the ability to maintain independence in daily life. In this regard, physical activity may constitute an important component of an active and healthy lifestyle in later life, particularly because it may be related to subjective health perceptions, social relationships, and engagement in daily and social activities. Evidence among active older adults has also indicated that higher levels of physical activity are associated with better self-perceived health, more frequent social relationships, and greater satisfaction with health and social care services (Parra-Rizo and Sanchís-Soler, 2023). In addition, aerobic and resistance exercise have been discussed as relevant non-pharmacological approaches for supporting cardiovascular and psychological health in older adults (Parra-Rizo and López-Marín, 2020).

However, important gaps remain in the existing literature. First, although previous studies have extensively examined the association between physical exercise and depressive symptoms, relatively less attention has been given to how health perceptions and positive psychological states may be statistically connected within the same explanatory model (Zhang and Jiang, 2023). Second, previous studies have examined potential mediators such as social support and health control beliefs (Xue et al., 2021), but the possible sequential relationship between perceived physical health and subjective wellbeing has received less systematic attention. Rather than treating self-rated physical health and subjective wellbeing as two independent and parallel mediators, the present study proposes that better perceived physical health may be statistically associated with greater subjective wellbeing, which may in turn be associated with fewer depressive symptoms. This proposed ordering is grounded in the theoretical view that physical health perceptions constitute an important foundation for maintaining functional independence, life satisfaction, and positive emotional experiences in later life. Importantly, however, because all variables were measured within the same cross-sectional survey wave, this ordering should be understood as a theoretically proposed and statistically tested pathway rather than a confirmed temporal sequence.

Self-rated physical health refers to individuals’ subjective evaluation of their overall health condition and has been widely examined as an indicator associated with mental health outcomes among older adults. Compared with objective medical indicators, self-rated health reflects not only physical functioning but also individuals’ perceptions and cognitive evaluations of their own health status. Previous studies have shown that physical exercise is associated with better physical functioning, balance ability, and cardiopulmonary fitness among older adults (Bai et al., 2022). Better perceived physical health may also be associated with fewer feelings of physical limitation and dependence, which may be relevant to depressive symptoms. Subjective wellbeing, which reflects individuals’ overall evaluation of life satisfaction and emotional experience, is another important psychological resource in later life. Existing evidence suggests that physical exercise is associated with subjective wellbeing through factors such as positive affect, self-efficacy, and social interaction (Toros et al., 2023; Gu and Liu, 2025). Higher subjective wellbeing is also associated with better emotional adjustment and lower levels of depressive symptoms. Taken together, these findings suggest that self-rated physical health and subjective wellbeing may represent interconnected pathways through which physical exercise is statistically associated with depressive symptoms among older adults.

Based on these considerations, this study used data from the 2023 Chinese General Social Survey (CGSS) to examine the association between physical exercise and depressive symptoms among older adults and to explore the potential indirect pathways involving self-rated physical health and subjective wellbeing. Specifically, a serial mediation model was constructed to examine whether self-rated physical health was statistically associated with subjective wellbeing in the pathway linking physical exercise and depressive symptoms. The study further compared the relative sizes of the specific indirect associations. By examining these theoretically proposed pathways within a single model, this study seeks to extend existing research beyond the simple exercise–depressive symptoms association and provide empirical evidence relevant to mental health promotion and public sports services in the context of active aging. Given the cross-sectional nature of the data, the proposed serial pathway is interpreted as a theoretical and statistical pathway rather than a causal or temporal mechanism.

## 2 Literature review and hypothesis development

### 2.1 Association between physical exercise and depressive symptoms

Depression is a common negative emotional experience among older adults, which may not only impair mental health but also contribute to frailty, malnutrition, and declines in social functioning. Existing studies have generally reported favorable associations between physical exercise and mental health outcomes among older adults. Some studies have indicated that depressive symptoms may reduce older adults’ motivation to participate in physical exercise (Miller et al., 2019). At the same time, a larger body of evidence has reported that regular physical exercise is associated with lower levels of depressive symptoms among older adults (De Sousa et al., 2021; Catalan-Matamoros et al., 2016). Physical exercise may also be related to positive emotional experiences, social interaction, self-efficacy, and perceived control over life, which may partly explain its association with depressive symptoms. Based on these findings, the following hypothesis was proposed:

*H1*: Physical exercise is negatively associated with depressive symptoms among older adults.

Previous studies have further shown that the health benefits of physical exercise are mainly reflected in two aspects: the promotion of physical health and the enhancement of psychological resources (Xu et al., 2024). Therefore, the effect of physical exercise on depression may not operate through a single pathway, but rather through the combined influence of improved physical health and the accumulation of positive psychological resources. Based on this perspective, the present study further introduced self-rated physical health and subjective wellbeing as mediating variables to explore their underlying mechanisms in the relationship between physical exercise and depression among older adults.

### 2.2 The potential mediating role of self-rated physical health in the association between physical exercise and depressive symptoms

Regular physical exercise can effectively improve physical health conditions among older adults, including enhancing cardiopulmonary function (Jia et al., 2018), improving physical functioning (Wang J. et al., 2026), reducing the risk of chronic diseases (Di Raimondo et al., 2016), and delaying cognitive decline (Chuang et al., 2023; Angevaren et al., 2008). According to health protection theory, good physical health conditions may reduce negative emotional experiences caused by functional limitations and disease burden, thereby lowering the risk of depression (Shi et al., 2024). In contrast, physical dysfunction, chronic pain, and declines in daily living abilities are more likely to increase negative emotional experiences and induce depressive symptoms (Ramírez-Fernández et al., 2018).

In addition, self-rated physical health reflects not only individuals’ objective health conditions, but also their subjective perceptions and evaluations of their own health status. Positive health perceptions may enhance individuals’ positive evaluations of life and reduce negative emotional experiences (Fox et al., 2026). Therefore, physical exercise may be associated with lower levels of depressive symptoms through its association with self-rated physical health. Based on this reasoning, the following hypothesis was proposed:

*H2*: Self-rated physical health mediates the relationship between physical exercise and depressive symptoms among older adults.

### 2.3 The potential mediating role of subjective wellbeing in the association between physical exercise and depressive symptoms

Subjective wellbeing refers to individuals’ overall positive psychological evaluations based on their life conditions, mainly including life satisfaction and positive affect (Diener et al., 1999). Existing studies have shown that physical exercise may be associated with more positive emotional experiences and higher subjective wellbeing through factors such as social interaction, self-efficacy, and sense of life meaning (Won et al., 2020). Higher levels of subjective wellbeing may strengthen individuals’ positive cognition and emotional regulation abilities (Liu et al., 2014), thereby reducing the risk of depression (Chao, 2016). Conversely, low levels of subjective wellbeing may contribute to the accumulation of negative emotions and increase the likelihood of depression (Zhao et al., 2022). Previous studies have also confirmed a significant negative association between subjective wellbeing and depressive symptoms (Wang W. et al., 2026). Based on these findings, the following hypothesis was proposed:

*H3*: Subjective well-being mediates the relationship between physical exercise and depressive symptoms among older adults.

### 2.4 The potential serial mediation pathway linking physical exercise, self-rated physical health, subjective wellbeing, and depressive symptoms

The theory of active aging provides a theoretical basis for considering physical health as an important condition associated with positive psychological functioning in later life. Physical exercise is associated with physical functioning and health perceptions among older adults (Hirosaki et al., 2013), which may in turn be related to participation in social activities and maintenance of independent living (Grönstedt et al., 2013). Better perceived physical health may also be associated with greater life satisfaction and more positive emotional experiences (Wróblewska et al., 2023; Grundberg et al., 2012), which are important components of subjective wellbeing (Garatachea et al., 2009; Gildner et al., 2019). Higher subjective wellbeing is, in turn, associated with lower levels of depressive symptoms (Li et al., 2025). Thus, from a theoretical perspective, self-rated physical health may precede subjective wellbeing in a proposed serial pathway linking physical exercise and depressive symptoms. This proposed ordering is based on theoretical considerations and the statistical structure of the mediation model; because the present data are cross-sectional, it should not be interpreted as evidence of temporal precedence or causality. Based on this theoretical framework, the following hypothesis was proposed:

*H4*: Self-rated physical health and subjective well-being show a serial indirect association in the relationship between physical exercise and depressive symptoms among older adults.

## 3 Methods

### 3.1 Participants

This study was based on data from the 2023 Chinese General Social Survey (CGSS). The CGSS adopts a multistage stratified sampling method covering 31 provinces, autonomous regions, and municipalities in China. The survey includes more than 400 variables related to sociodemographic characteristics, housing, health, migration, lifestyle, social attitudes, class identity, labor market participation, social security, and family conditions, with relatively high data quality and national representativeness. To focus on the older adult population, this study restricted the sample to respondents aged 60 years and above. After excluding cases with missing values and abnormal data, a total of 2,233 valid samples were included in the final analysis. Sociodemographic variables included gender, age, marital status, educational level, household registration type, number of children, and self-rated economic status. The sociodemographic characteristics of the participants are presented in Table 1.

**Table 1:** Sociodemographic characteristics of the participants.

Variables	Measurement/coding	Mean	SD
Gender	Male = 1, female = 0	0.471	0.499
Age	Actual age of respondents at the time of survey	69.808	6.775
Marital status	Married (including remarried and separated but legally married) = 1; unmarried, divorced, widowed, or cohabiting = 0	0.710	0.454
Education level	Primary school or below = 1, junior high school = 2, senior high school/technical secondary school = 3, college or above = 4	1.826	0.930
Hukou status	Non-agricultural hukou = 1, agricultural hukou = 0	0.446	0.497
Number of children	No child = 0, one child = 1, two or more children = 2	1.655	0.517
Self-rated economic status	Lower = 1, lower-middle = 2, middle = 3, upper-middle = 4, upper = 5	1.504	0.770

SD, standard deviation.

### 3.2 Measurements

#### 3.2.1 Dependent variable: depressive symptoms

Depressive symptoms were assessed using a single self-reported CGSS item: “During the past 4 weeks, how often have you felt depressed or emotionally down?” Responses included “never,” “rarely,” “sometimes,” “often,” and “always,” which were coded from 1 to 5, respectively. Higher scores indicated higher levels of depressive symptoms. Because this measure consists of a single self-reported item rather than a validated multi-item clinical depression scale, it is intended to capture the frequency of depressive symptoms rather than diagnose clinical depression. Accordingly, the findings should be interpreted in terms of depressive symptoms rather than clinical depression.

#### 3.2.2 Independent variable: physical exercise

Physical exercise participation was measured using the CGSS item: “In the past year, have you regularly participated in physical exercise during your leisure time?” The item was assessed using a five-point Likert scale: “every day” = 5, “several times a week” = 4, “several times a month” = 3, “several times a year or less” = 2, and “never” = 1. Higher scores indicated more frequent participation in physical exercise. This measure captures the frequency of participation but does not provide information on exercise type, intensity, duration, or participation context. Therefore, the measure reflects frequency of exercise participation rather than a comprehensive assessment of physical exercise behavior.

#### 3.2.3 Control variables

To reduce potential confounding effects, this study controlled for variables from three dimensions: sociodemographic characteristics, physical health conditions, and psychosocial factors.

#### 3.2.4 Physical health conditions

Physical health conditions included self-rated physical health and body mass index (BMI). Self-rated physical health was measured using the question: “How would you rate your current physical health condition?” Responses ranged from “very unhealthy” to “very healthy” and were coded from 1 to 5, with higher scores indicating better perceived physical health.

BMI was calculated based on respondents’ self-reported height and weight. According to the Chinese Nutrition Society (2022) and the Guidelines for Appropriate BMI Range and Weight Management in Older Chinese Adults (T/CNSS 021-2023), BMI was categorized as follows: 18.5–23.9 kg/m^2^ for individuals aged 60–64 years, 20–26.9 kg/m^2^ for those aged 65–80 years, and 22–26.9 kg/m^2^ for those aged above 80 years. Respondents whose BMI fell within the recommended range were coded as 1; otherwise, they were coded as 0.

#### 3.2.5 Psychosocial factors

Drawing on previous CGSS-based studies (Hu et al., 2025; Yin and Liu, 2020; Xu et al., 2023), this study incorporated subjective wellbeing, social security status, perceived social fairness, subjective social class, social interaction, and social trust into the statistical model as psychosocial control variables. These variables reflected the social environment and individual cognitive evaluations associated with older adults’ mental health. Among them, social interaction and social trust represented social integration and the quality of social relationships, whereas subjective wellbeing and perceived social fairness reflected individuals’ overall emotional and cognitive evaluations of their life circumstances and social status (Kawachi and Berkman, 2001). Controlling for these variables helped reduce potential confounding bias and allowed for a more accurate estimation of the net effect of physical exercise on depressive symptoms among older adults. Subjective wellbeing was measured using a commonly adopted approach in Chinese wellbeing research through the item: “Overall, do you feel that your life is happy?” Responses ranged from “very unhappy” to “very happy” and were coded from 1 to 5, with higher scores indicating higher levels of subjective wellbeing.

### 3.3 Data analysis

All statistical analyses were conducted using IBM SPSS Statistics and the PROCESS macro developed by Hayes (2018). The analytical procedure consisted of the following steps:

Common method bias test. Because the data obtained from the Chinese General Social Survey were based on self-reported responses, a common method bias test was conducted to examine potential common method variance. Harman’s single-factor test was conducted as an exploratory assessment. The first unrotated factor accounted for 24.23% of the total variance, which was below the commonly used 40% reference value (Tang and Wen, 2020). This result did not indicate a dominant single-factor pattern in the data. However, because Harman’s single-factor test has limited sensitivity for detecting common method variance, this result should not be interpreted as demonstrating that common method bias was completely absent.

Descriptive statistics. Means and standard deviations of the dependent variable, independent variable, and control variables were calculated to describe the sample characteristics.

Multiple linear regression analysis. To examine the association between physical exercise and depressive symptoms among older adults, depressive symptoms were treated as the dependent variable and physical exercise as the independent variable. Demographic characteristics, physical health status, and psychosocial factors were included as control variables. Hierarchical multiple linear regression models were employed by gradually adding different groups of control variables. Model 1 included only sociodemographic variables; Model 2 additionally incorporated physical health variables; Model 3 further added psychosocial variables; and Model 4 simultaneously included all control variables and the core explanatory variable, physical exercise. Robust standard errors were used in the regression models to reduce sensitivity to potential heteroscedasticity.

Correlation analysis. Pearson correlation analyses were performed among the core variables to preliminarily assess the direction and strength of their associations.

Mediation analysis. Based on both the statistical results and theoretical expectations, a serial mediation model was constructed to further explore the potential indirect pathways linking physical exercise and depressive symptoms among older adults. Specifically, the model aimed to examine the independent mediating effects of self-rated physical health and subjective wellbeing, as well as their serial mediating mechanism linking physical exercise and depressive symptoms. The PROCESS macro Model 6 developed by Andrew F. Hayes was employed to test the serial mediation effects. A bias-corrected nonparametric percentile Bootstrap method with 5,000 resamples was used to estimate the indirect effects and their 95% confidence intervals (CIs). A mediating effect was considered statistically significant when the 95% CI did not include zero.

Methodological consideration for ordinal variables. The core variables in this study were measured using five-category ordinal response scales. In the primary analyses, these scores were treated as approximately continuous and analyzed using linear regression and PROCESS-based mediation models. This approach was adopted because the variables contained five ordered categories, the sample size was relatively large (*N* = 2,233), and the primary objective was to estimate differences in observed scale scores and indirect associations rather than category-specific probabilities. Robust standard errors were used in the regression models to reduce sensitivity to potential heteroscedasticity. Nevertheless, because the measures are ordinal single-item variables, the estimated coefficients should be interpreted as statistical associations on the observed response scales rather than as causal effects or changes in the probability of belonging to a specific response category.

## 4 Results

### 4.1 Descriptive statistics of the study variables

Table 2 reports the descriptive statistics of the study variables, including depressive symptoms, physical exercise, self-rated physical health, subjective wellbeing, and the main control variables.

**Table 2:** Descriptive statistics of the study variables.

Variables	Measurement/coding	Mean	SD
Dependent variable			
Depressive symptoms	Never = 1, rarely = 2, sometimes = 3, often = 4, always = 5	1.255	1.198
Independent variable			
Physical exercise	Never = 1, several times a year or less = 2, several times a month = 3, several times a week = 4, every day = 5	2.521	1.745
Physical health variables			
Self-rated physical health	Very unhealthy = 1, relatively unhealthy = 2, average = 3, relatively healthy = 4, very healthy = 5	2.983	1.129
BMI status	Normal BMI = 1, abnormal BMI = 0	0.509	0.500
Psychosocial variables			
Subjective wellbeing	Very unhappy = 1, relatively unhappy = 2, neutral = 3, relatively happy = 4, very happy = 5	3.910	0.897
Social security participation	Participation in at least one social insurance program = 1, no participation = 0	0.952	0.214
Perceived social fairness	Completely unfair = 1, relatively unfair = 2, neutral = 3, relatively fair = 4, completely fair = 5	3.341	1.106
Social class	Self-rated social class ranging from 0 to 10 (low to high)	4.256	1.979
Social interaction	Self-rated social interaction ranging from 0 to 10 (low to high)	4.013	3.458
Social trust	Strongly disagree = 1, relatively disagree = 2, neutral = 3, relatively agree = 4, strongly agree = 5	3.596	1.019

SD, standard deviation; BMI, body mass index.

### 4.2 The association between physical exercise and depressive symptoms among older adults

Model 1 included only demographic characteristics. The results indicated that gender (*β* = −0.185, *p* < 0.001), educational attainment (*β* = −0.160, *p* < 0.001), household registration type (*β* = −0.224, *p* < 0.001), and self-rated economic status (*β* = −0.323, *p* < 0.001) were significantly associated with depressive symptoms among older adults.

Model 2 added physical health variables based on Model 1. The results showed that self-rated physical health had a significant negative association with depressive symptoms (*β* = −0.439, *p* < 0.001). Meanwhile, the adjusted *R*^2^ increased from 0.106 to 0.264, indicating that physical health status was an important factor in explaining depressive symptoms among older adults, with self-rated physical health emerging as a significant predictor.

Model 3 further incorporated psychosocial variables. Among these variables, subjective wellbeing (*β* = −0.215, *p* < 0.001) and social security status (*β* = −0.202, *p* < 0.05) showed significant negative associations with depressive symptoms. The adjusted *R*^2^ further increased from 0.264 to 0.290, suggesting that psychosocial factors also played an important role in explaining depressive symptoms among older adults. In particular, subjective wellbeing appeared to be the strongest psychosocial predictor of depressive symptoms.

Model 4 added the core independent variable, physical exercise, to all control variables. The results demonstrated that physical exercise had a significant negative association with depressive symptoms (*β* = −0.040, *p* < 0.01), indicating that higher frequencies of participation in physical exercise were associated with lower levels of depressive symptoms among older adults. Specifically, after controlling for other variables, each one-level increase in physical exercise frequency was associated with an average decrease of 0.04 points in depressive symptom scores. The adjusted *R*^2^ of Model 4 was 0.293, indicating relatively stable explanatory power of the model for depressive symptoms among older adults.

Overall, these findings suggest that physical exercise was independently and negatively associated with depressive symptoms among older adults. Even after controlling for demographic characteristics, physical health status, and psychosocial factors, the association remained statistically significant. Therefore, Hypothesis H1 was supported (Table 3).

**Table 3:** Multiple linear regression analysis of factors influencing depressive symptoms among older adults.

Variables	Model 1	Model 2	Model 3	Model 4
Sample size	2,233	2,233	2,233	2,233
Constant	2.318*** (0.280)	3.698*** (0.263)	4.043*** (0.290)	4.092*** (0.290)
Gender	−0.185*** (0.049)	−0.114* (0.045)	−0.107* (0.044)	−0.100* (0.044)
Age	−0.003 (0.004)	−0.007* (0.003)	−0.004 (0.003)	−0.005 (0.003)
Marital status	−0.035 (0.056)	−0.058 (0.051)	−0.058 (0.050)	−0.063 (0.050)
Education level	−0.160*** (0.031)	−0.131*** (0.028)	−0.129*** (0.027)	−0.120*** (0.028)
Hukou status	−0.224*** (0.059)	−0.135* (0.054)	−0.131* (0.053)	−0.099 (0.054)
Number of children	0.064 (0.053)	0.031 (0.048)	0.047 (0.047)	0.047 (0.047)
Self-rated economic status	−0.323*** (0.032)	−0.193*** (0.030)	−0.134*** (0.032)	−0.132*** (0.031)
Self-rated physical health		−0.439*** (0.020)	−0.404*** (0.020)	−0.397*** (0.020)
BMI status		0.017 (0.044)	0.007 (0.043)	0.006 (0.043)
Subjective wellbeing			−0.215*** (0.027)	−0.208*** (0.027)
Social security participation			−0.202* (0.101)	−0.212* (0.101)
Perceived social fairness			−0.022 (0.022)	−0.025 (0.022)
Social class			−0.003 (0.012)	−0.003 (0.012)
Social interaction			−0.006 (0.006)	−0.004 (0.006)
Social trust			−0.009 (0.022)	−0.006 (0.022)
Physical exercise				−0.040** (0.013)
Adjusted *R*^2^	0.106	0.264	0.290	0.293

Robust standard errors are reported in parentheses. *p < 0.05, **p < 0.01, ***p < 0.001.

### 4.3 Potential indirect pathways linking physical exercise and depressive symptoms among older adults

The above findings indicated that self-rated physical health and subjective wellbeing were the most important predictors of depressive symptoms among older adults within the domains of physical health and psychosocial factors.

#### 4.3.1 Correlation analysis

Before testing the serial mediation model, Pearson correlation analyses were conducted among the core variables. The results are presented in Table 4. Physical exercise was significantly negatively correlated with depressive symptoms (*r* = −0.215, *p* < 0.001), and significantly positively correlated with self-rated physical health (*r* = 0.215, *p* < 0.001) and subjective wellbeing (*r* = 0.152, *p* < 0.001). Self-rated physical health was significantly negatively correlated with depressive symptoms (*r* = −0.470, *p* < 0.001), and subjective wellbeing was also significantly negatively correlated with depressive symptoms (*r* = −0.297, *p* < 0.001). These correlations were consistent with the theoretical expectations and provided preliminary support for the subsequent serial mediation analysis.

**Table 4:** Correlation analysis of key variables.

Variables	Depressive symptoms	Physical exercise	Self-rated physical health	Subjective wellbeing
Depressive symptoms	–			
Physical exercise	−0.215***	–		
Self-rated physical health	−0.470***	0.215***	–	
Subjective wellbeing	−0.297***	0.152***	0.223***	–

***p < 0.001.

#### 4.3.2 Serial mediation model analysis

After controlling for the relevant variables, a serial mediation model was constructed with physical exercise as the independent variable, self-rated physical health and subjective wellbeing as mediators, and depressive symptoms as the dependent variable. The estimated path coefficients of the serial mediation model are presented in Figure 1, and the bootstrap results for the indirect associations are shown in Table 5.

**Figure 1:**
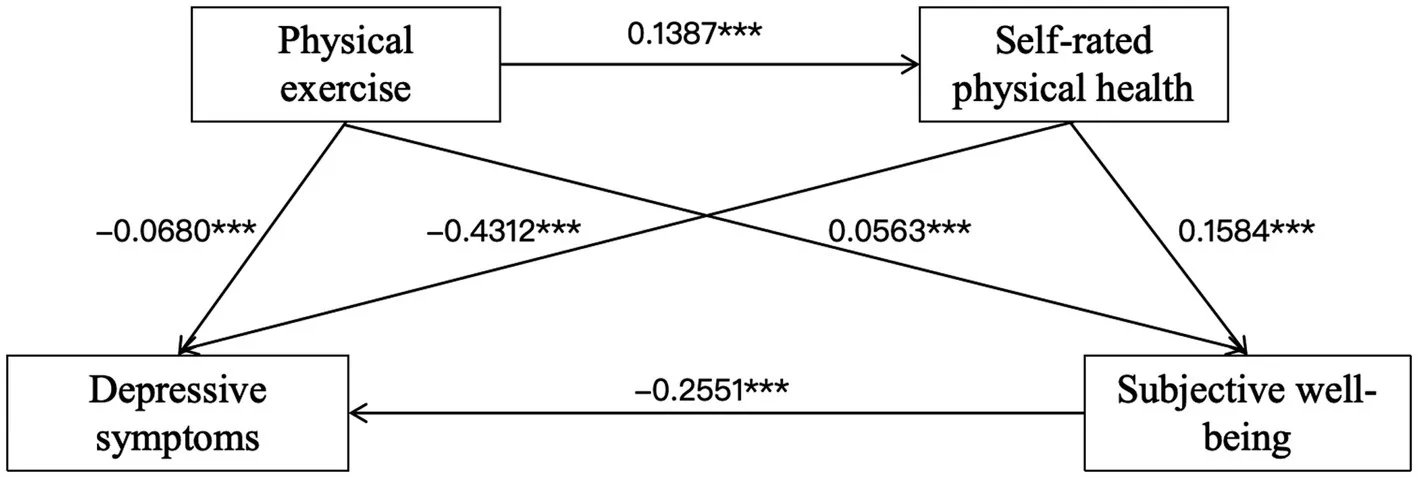
Potential serial mediation pathway linking physical exercise, self-rated physical health, subjective wellbeing, and depressive symptoms among older adults. *** *p* < 0.001.

**Table 5:** Bootstrap analysis of mediating effects.

Mediation pathway	Effect value	Boot SE	95% CI Lower	95% CI Upper	Relative mediating effect (%)
Total indirect effect of physical exercise on depressive symptoms	−0.0798	0.0073	−0.0945	−0.0656	54.0%
Physical exercise → Self-rated physical health → Depressive symptoms	−0.0598	0.0064	−0.0729	−0.0478	40.5%
Physical exercise → Subjective wellbeing → Depressive symptoms	−0.0144	0.0031	−0.0205	−0.0087	9.7%
Physical exercise → Self-rated physical health → Subjective wellbeing → Depressive symptoms	−0.0056	0.0010	−0.0077	−0.0037	3.8%

Bootstrap samples = 5,000; CI, confidence interval. Indirect effects are considered significant when the 95% confidence interval does not include zero.

The path analysis results indicated that physical exercise significantly positively associated with self-rated physical health (*β* = 0.1387, *p* < 0.001), and self-rated physical health significantly positively associated with subjective wellbeing (*β* = 0.1584, *p* < 0.001). In turn, subjective wellbeing significantly negatively associated with depressive symptoms (*β* = −0.2551, *p* < 0.001). Meanwhile, self-rated physical health also had a significant direct negative effect on depressive symptoms (*β* = −0.4312, *p* < 0.001), indicating that self-rated physical health represented a significant indirect pathway linking physical exercise and depressive symptoms.

To further verify the mediating roles of self-rated physical health and subjective wellbeing, the bias-corrected nonparametric percentile Bootstrap method was used to estimate the significance of the specific indirect effects. As shown in Table 5, the 95% CI for the serial mediation pathway of “physical exercise → self-rated physical health → subjective wellbeing → depressive symptoms” was [−0.0077, −0.0037]. The 95% CI for the indirect effect through self-rated physical health alone was [−0.0729, −0.0478], whereas the 95% CI for the indirect effect through subjective wellbeing alone was [−0.0205, −0.0087]. Because none of the 95% confidence intervals included zero, all three indirect associations were statistically significant. These findings provide statistical support for the hypothesized indirect pathways, but they should not be interpreted as evidence of causal mediation or temporal ordering given the cross-sectional design.

Further comparison of the estimated indirect associations showed that the total association between physical exercise and depressive symptoms was −0.148, with a direct association of −0.068 (46.0% of the total association) and a total indirect association of −0.080 (54.0% of the total association). The three specific indirect associations were −0.060, −0.014, and −0.006, accounting for 40.5, 9.7, and 3.8% of the total association, respectively. The total explained variance of the serial mediation model was 0.293. Among this, the direct pathway from physical exercise to depressive symptoms accounted for 46.0% of the total effect, whereas the indirect pathways accounted for 54.0% of the total effect. Therefore, the proposed serial mediation model was supported, and Hypotheses H2, H3, and H4 were confirmed.

Overall, the findings indicate that physical exercise was associated with lower levels of depressive symptoms both directly and indirectly through self-rated physical health and subjective wellbeing. The results also provided statistical support for the proposed serial pathway involving self-rated physical health and subjective wellbeing. These findings identify several potential indirect pathways that may help explain the observed association between physical exercise and depressive symptoms among older adults. However, these pathways should be interpreted as statistical and theoretical pathways rather than confirmed causal mechanisms.

## 5 Discussion

Using data from the 2023 Chinese General Social Survey (CGSS), this study employed multiple linear regression and a serial mediation model to examine the association between physical exercise and depressive symptoms among older adults and to explore potential indirect pathways involving self-rated physical health and subjective wellbeing. The findings indicated that: (1) higher frequency of physical exercise was associated with lower levels of depressive symptoms among older adults; (2) physical exercise showed significant indirect associations with depressive symptoms through self-rated physical health and subjective wellbeing, respectively, as well as through the proposed serial pathway of “self-rated physical health → subjective wellbeing”; and (3) among the three specific indirect pathways examined, the indirect association through self-rated physical health was the largest, followed by that through subjective wellbeing, whereas the serial indirect association was smaller but statistically significant. These findings should be interpreted as cross-sectional statistical associations rather than causal effects or confirmed temporal mechanisms.

### 5.1 Association between physical exercise and depressive symptoms among older adults

The present study found that, even after controlling for demographic characteristics, physical health status, and psychosocial factors, physical exercise was still significantly and negatively associated with depressive symptoms among older adults. This finding is consistent with previous studies (De Sousa et al., 2021; Zhang et al., 2024). From a theoretical perspective, several mechanisms proposed in previous literature may help contextualize the observed association between physical exercise and depressive symptoms.

First, from a physiological perspective, regular physical exercise can promote the release of neurotransmitters such as endorphins and dopamine (Hossain et al., 2024), which are associated with mood enhancement and reductions in anxiety and depressive symptoms. Second, from a psychological perspective, physical exercise may provide older adults with a stronger sense of purpose, achievement, and self-efficacy (O'Neil-Pirozzi, 2021), thereby interrupting the cycle of negative emotions caused by retirement, widowhood, empty-nest experiences, and other adverse life events. Third, from a sociological perspective, physical exercise may offer older adults opportunities to maintain regular daily routines and continue social role engagement, which may help sustain positive psychological states (Yi et al., 2025). These physiological, psychological, and sociological explanations are derived from previous literature and were not directly measured or tested in the present study; therefore, they should be regarded as possible explanations rather than mechanisms demonstrated by the current data.

Therefore, physical exercise may be a relevant behavioral resource for physical and psychological wellbeing among older adults, although its causal role in preventing depressive symptoms cannot be established from the present cross-sectional data.

### 5.2 The potential mediating role of self-rated physical health in the association between physical exercise and depressive symptoms

This study found a significant indirect association between physical exercise and depressive symptoms through self-rated physical health. The estimated indirect association was −0.060, accounting for 40.5% of the total association, which was the largest among the three specific indirect pathways examined in the model. This finding was consistent with Hypothesis H2.

Several explanations may account for this result. First, from the perspective of exercise mechanisms, physical exercise has been associated with better physical health status. Among older adults, physical exercise may enhance self-rated physical health by improving cardiopulmonary function, increasing muscle strength and balance ability, and reducing the risk of chronic diseases (Bai et al., 2022). Second, physical exercise contributes to both physical and psychological wellbeing. Improvements in physical health may enable older adults to perform daily activities more independently, reduce reliance on others, and alleviate feelings of helplessness, physical discomfort, and life restrictions associated with physical decline (Grundberg et al., 2012). These positive bodily experiences may be transformed into more positive self-evaluations through cognitive appraisal processes, thereby reducing depressive symptoms.

Furthermore, from a cultural perspective, the emphasis placed on health in Chinese society may provide a possible contextual explanation for the observed association between self-rated physical health and depressive symptoms. For example, the commonly expressed belief that “health is the greatest wealth” may reflect the importance attached to physical health in later life. However, cultural beliefs were not directly measured in the present study; therefore, this interpretation should be regarded as a possible cultural explanation rather than an empirically tested mechanism.

It is also noteworthy that this study employed self-rated physical health rather than objective medical indicators as the measurement tool. This finding highlights the importance of subjective health perceptions in emotional regulation among older adults. The present study therefore suggests that, within the statistical model examined here, self-rated physical health showed a substantial indirect association with depressive symptoms. However, because objective medical indicators were not directly compared with self-rated physical health in this study, no conclusion can be drawn regarding whether self-rated health is more predictive of mental health outcomes than objective health indicators.

### 5.3 The potential mediating role of subjective wellbeing in the association between physical exercise and depressive symptoms

The present study also found a significant indirect association between physical exercise and depressive symptoms through subjective wellbeing. The estimated indirect association was −0.014, accounting for 9.7% of the total association. This finding was consistent with Hypothesis H3.

Subjective wellbeing refers to individuals’ overall positive evaluations of their quality of life based on personal standards and generally includes life satisfaction, positive affect, and negative affect. The mechanisms proposed to explain the association between physical exercise and subjective wellbeing among older adults appear to be multifaceted.

From a physiological perspective, exercise promotes the release of endorphins and dopamine, thereby generating immediate pleasurable experiences (Hossain et al., 2024). From a psychological perspective, physical exercise may strengthen self-efficacy while providing individuals with a greater sense of purpose and accomplishment (Toros et al., 2023). From a social perspective, physical exercise is often accompanied by social interaction, which may alleviate loneliness (Gu and Liu, 2025). These explanations are based on previous theoretical and empirical literature, whereas the present study directly tested only the statistical associations among physical exercise, subjective wellbeing, and depressive symptoms.

Enhanced subjective wellbeing may be associated with depressive symptoms through several pathways. First, individuals with higher subjective wellbeing tend to experience more positive emotions (Liu et al., 2014). These positive emotional experiences may strengthen self-worth and perceived life control, foster positive cognitive biases, and suppress the development of depressive symptoms (Chao, 2016). Second, increased subjective wellbeing may help interrupt cycles of negative emotions and reduce excessive rumination on adverse events. Third, subjective wellbeing itself may function as a psychological resilience resource, buffering the negative effects of stressful life events on mental health.

The mediating effect of subjective wellbeing was smaller than that of self-rated physical health in this study. One possible explanation is that subjective wellbeing is a relatively stable psychological trait influenced by multiple factors, such as personality characteristics, social support, and economic conditions. Therefore, the positive influence of physical exercise on subjective wellbeing may require long-term accumulation to become fully apparent. In contrast, improvements in physical health may be perceived more directly and strongly among older adults. One possible explanation is that self-rated physical health may be more closely connected with daily functional conditions and perceived physical limitations in older adults, whereas subjective wellbeing may reflect a broader psychological evaluation influenced by multiple factors. However, these explanations were not directly tested in the present study and should therefore be interpreted cautiously.

### 5.4 The potential serial mediation pathway linking physical exercise and depressive symptoms among older adults

This study further identified a statistically significant indirect association between physical exercise and depressive symptoms through the serial pathway of “self-rated physical health → subjective well-being.” This finding was consistent with Hypothesis H4 and provides statistical support for the theoretically proposed ordering of the two mediators. However, because all variables were measured at the same survey wave, the analysis cannot establish that physical exercise temporally precedes changes in self-rated physical health, subjective wellbeing, and depressive symptoms. The serial mediation pathway is statistically consistent with the proposed sequence from self-rated physical health to subjective wellbeing and depressive symptoms, but this statistical ordering should not be interpreted as a confirmed temporal process.

One possible explanation is that physical exercise first improves physical health status, including increased vitality, reduced physical discomfort, and enhanced mobility. These positive bodily changes may provide older adults with more stable physiological resources and greater functional capacity (Asp et al., 2025). Improved physical health may further enable older adults to participate more actively in daily activities, maintain independent living, and reduce reliance on caregiving from others. These positive physical experiences may subsequently enhance life satisfaction and positive emotional experiences, thereby improving subjective wellbeing. Enhanced subjective wellbeing may ultimately be associated with fewer depressive symptoms.

Taken together with previous literature, the findings can be discussed within a tentative “physiological → psychological → emotional” framework. At the physiological level, physical exercise may be associated with better physical functioning and self-rated health. At the psychological level, previous research suggests that exercise may be related to self-efficacy, positive affect, and subjective wellbeing. At the emotional level, higher subjective wellbeing is associated with lower depressive symptoms. However, these physiological and psychological processes were not directly measured in the present study. Therefore, this framework should be regarded as a theoretical interpretation that requires further longitudinal or experimental testing rather than as a mechanism demonstrated by the current data.

Importantly, the physiological and psychological mechanisms do not operate independently but rather interact with one another. For example, improved physical health may enhance subjective wellbeing, whereas increased subjective wellbeing may further motivate individuals to maintain healthy behaviors, thereby forming a positive feedback loop. Future studies are needed to further investigate these dynamic interactions.

### 5.5 Comparison and integration of the three mediating pathways

Using the Bootstrap estimates, this study compared the relative sizes of the three specific indirect associations and found that the indirect association through self-rated physical health was the largest (40.5%), followed by the indirect association through subjective wellbeing (9.7%), whereas the serial indirect association was smaller (3.8%). Because the mediators were measured using different single-item ordinal scales, the relative magnitudes of the indirect estimates should be interpreted cautiously. The percentages reported here describe the proportion of the estimated total association accounted for by each indirect pathway within this model and should not be interpreted as evidence that self-rated physical health is universally a stronger predictor or mediator than subjective wellbeing.

Several explanations may account for these findings. First, the relatively larger estimated indirect association through self-rated physical health within the present model may reflect that perceived physical health among older adults is closely associated with basic aspects of quality of life, including self-care ability, independence, and physical comfort. For older adults, the traditional Chinese belief that “health is the greatest wealth” represents not merely a cultural expression but also a realistic reflection of daily life. When older adults perceive positive changes in their physical condition through exercise, these changes may rapidly and directly translate into more positive life evaluations, thereby alleviating depressive symptoms. In addition, the physical benefits of exercise—such as increased muscle strength, improved joint function, enhanced cardiopulmonary capacity, and reduced chronic disease symptoms—may be perceived more directly and strongly among older adults, contributing to the larger mediating effect.

Second, the independent mediating effect of subjective wellbeing ranked second because subjective wellbeing is a relatively stable psychological trait influenced by multiple factors, including personality, social support, and economic conditions. Consequently, the positive impact of physical exercise on subjective wellbeing may require long-term accumulation before becoming fully evident. However, once a high level of subjective wellbeing is established, its protective effect against depressive symptoms may be enduring and profound. Therefore, interventions targeting depression among older adults should focus not only on short-term improvements in physical health but also on the long-term cultivation of subjective wellbeing.

Finally, although the serial mediating effect was relatively small, it remained statistically significant, indicating a progressive relationship between physical health and subjective wellbeing. This finding suggests that simply improving subjective wellbeing without enhancing physical health, or merely improving physical health without facilitating psychological transformation, may not fully maximize the potential mental health benefits associated with physical exercise. From a practical perspective, the findings suggest that programs promoting physical activity among older adults may benefit from considering both perceived physical health and subjective wellbeing. However, whether changes in these factors causally mediate the relationship between exercise and depressive symptoms should be examined using longitudinal or experimental designs.

### 5.6 Limitations

Several limitations should be acknowledged. First, the cross-sectional design of this study limits causal inference, and the observed indirect pathways should not be interpreted as causal mechanisms. Longitudinal or experimental studies are needed to further examine the temporal relationships among physical exercise, self-rated physical health, subjective wellbeing, and depressive symptoms. Second, depressive symptoms were assessed using a single self-reported item, rather than a validated multi-item scale or clinical assessment. Therefore, the findings should be interpreted as relating to depressive symptoms rather than clinical depression. In addition, physical exercise was measured only by frequency, without capturing exercise type, intensity, duration, or context, which may influence psychological outcomes differently. Third, most variables were obtained through self-reported measures collected in the same survey, which may introduce self-report bias and common method variance. Although Harman’s single-factor test did not indicate a dominant single factor, this approach cannot completely rule out common method bias. Future studies should combine subjective measures with objective indicators or multi-source data. Finally, although several potential confounders were controlled, residual confounding cannot be excluded. Moreover, physiological, psychological, social, and cultural mechanisms discussed in previous literature were not directly measured in this study and should be further investigated using longitudinal or experimental designs.

## 6 Conclusion

Based on data from the 2023 Chinese General Social Survey (CGSS), this study examined the association between physical exercise and depressive symptoms among older adults and explored potential indirect pathways involving self-rated physical health and subjective wellbeing. The findings showed that higher frequency of physical exercise was associated with lower levels of depressive symptoms. Physical exercise also showed significant indirect associations with depressive symptoms through self-rated physical health (40.5%) and subjective wellbeing (9.7%), as well as through the proposed serial pathway of “self-rated physical health → subjective wellbeing” (3.8%). The total indirect association accounted for 54.0% of the estimated total association, compared with 46.0% for the direct association. Among the three specific indirect pathways, the pathway involving self-rated physical health showed the largest estimated indirect association within the present model.

These findings provide statistical support for the proposed associations among physical exercise, self-rated physical health, subjective wellbeing, and depressive symptoms and suggest that health perceptions and positive psychological wellbeing may be relevant to understanding the relationship between physical exercise and mental health in later life. However, because the study used cross-sectional data, the observed associations and indirect pathways cannot establish causality or confirm temporal ordering. In particular, reverse causality is possible, as older adults with fewer depressive symptoms may be more likely to engage in physical exercise, perceive their health more positively, and report higher subjective wellbeing. Therefore, the proposed serial pathway should be regarded as a theoretical and statistical pathway that requires validation in longitudinal or experimental research.

## Data Availability

Publicly available datasets were analyzed in this study. This data can be found here: http://cgss.ruc.edu.cn/index.htm.
